# Crude Extracts of *Caenorhabditis elegans* Suppress Airway Inflammation in a Murine Model of Allergic Asthma

**DOI:** 10.1371/journal.pone.0035447

**Published:** 2012-04-25

**Authors:** Sung Eun Kim, Jae-Hwan Kim, Byung-Hoon Min, Young Mee Bae, Sung-Tae Hong, Min-Ho Choi

**Affiliations:** 1 Department of Parasitology and Tropical Medicine, Seoul National University College of Medicine, and Institute of Endemic Diseases, Seoul National University Medical Research Center, Seoul, Korea; 2 Department of Medicine, Samsung Medical Center, Sungkyunkwan University School of Medicine, Seoul, Korea; Trinity College Dublin, Ireland

## Abstract

Epidemiological studies suggest an inverse relationship between helminth infections and allergic disease, and several helminth-derived products have been shown to suppress allergic responses in animals. This study was undertaken to evaluate the effect of a crude extract of *Caenorhabditis elegans* on allergic airway inflammation in a murine model of asthma. Allergic airway inflammation was induced in BALB/c mice by sensitization with ovalbumin. The effect of the *C. elegans* crude extract on the development of asthma and on established asthma was evaluated by analyzing airway hyperresponsiveness, serum antibody titers, lung histology and cell counts and cytokine levels in the bronchoalveolar lavage fluid. The role of IFN-γ in the suppression of asthma by the *C. elegans* crude extract was investigated in IFN-γ knockout and wild-type mice. When mice were sensitized with ovalbumin together with the crude extract of *C. elegans*, cellular infiltration into the lung was dramatically reduced in comparison with the ovalbumin-treated group. Treatment of mice with the *C. elegans* crude extract significantly decreased methacholine-induced airway hyperresponsiveness and the total cell counts and levels of IL-4, IL-5 and IL-13 in the bronchoalveolar lavage fluid but increased the levels of IFN-γ and IL-12. Sensitization with the *C. elegans* crude extract significantly diminished the IgE and IgG1 responses but provoked elevated IgG2a levels. However, the suppressive effect of the *C. elegans* crude extract was abolished in IFN-γ knockout mice, and the Th2 responses in these mice were as strong as those in wild-type mice sensitized with ovalbumin. The crude extract of *C. elegans* also suppressed the airway inflammation associated with established asthma. This study provides new insights into immune modulation by the *C. elegans* crude extract, which suppressed airway inflammation in mice not only during the development of asthma but also after its establishment by skewing allergen-induced Th2 responses to Th1 responses.

## Introduction

The incidence of allergic diseases such as asthma, allergic rhinitis and eczema has steadily increased during the recent decades, especially in developed countries or urban areas of developing countries where helminth infections are rare or under control [1], [2]. Although allergic diseases and helminth infections both illicit Th2 responses, helminths have been known to provoke anti-inflammatory responses rather than allergic reactions in humans and animals [1], [3], [4]. A significant amount of epidemiological evidence from human field studies has suggested the existence of an inverse relationship between helminth infections and asthma and allergic sensitization [5]–[8]. However, other studies have reported no protective effects or enhanced allergic sensitization in individuals infected with parasites [9]–[11].

Experimental studies using animal models have also shown varying effects of parasite infection on the protection of the host against airway inflammation and allergic disease [1]. Infection with *Strongyloides stercoralis* or *Nippostrongylus brasiliensis* in mice suppressed experimental airway inflammation [12], [13], whereas *Toxocara canis* infection exacerbated the allergic responses to ovalbumin (OVA) in mice [14]. *Schistosoma mansoni* infection in mice caused different responses to an allergen depending on the production of eggs within the host; chronic infection with male and female worms aggravated OVA-induced airway hyperresponsiveness (AHR), but experimental infection with male schistosomes only protected mice from AHR [15]. This conflicting association between helminth infections and allergic diseases may be the result of several factors, including the species of parasite, the worm burden, the frequency and duration of infection and the timing of infection [9], [14].

Recently, helminth therapy has been used to ameliorate allergic or inflammatory diseases [16]–[19], and studies have reported promising outcomes, especially in the treatment of inflammatory bowel disease [18], [19]. However, the use of helminths for the treatment of inflammatory diseases has several potential side effects, including iatrogenic infection, general immune suppression, anaphylactic or atopic reactions and cross-reactivity with allergens [1]. Additional limitations of helminth therapy may include the difficulty of preparing specific pathogen-free eggs or larvae, the high cost of the therapy and poor patient compliance with consuming eggs or worms as therapeutic agents. An alternative solution to overcome these prospective problems would be the use of helminth-derived products that have anti-allergic or anti-inflammatory properties [16]. Several helminth-derived products that are known to alter the immune responses of the host and to have therapeutic potential for inflammatory diseases have been suggested based on data from animal models of human diseases [1], [16], [20].

Asthma is a complex disorder associated with Th2 immune responses directed to allergens and is characterized by airway inflammation, AHR, variable airflow obstruction and airway remodeling [21]–[23]. The mainstay of asthma treatment consists of inhaled or oral corticosteroids and long-acting β2-adrenoceptor agonists; however, these treatments are not curative, and symptoms return soon after treatment termination [21]. Reducing or eliminating allergen-specific Th2 responses in the early stage of asthma may lead to disease remission, which suggests that this may be one potential strategy for the development of new drugs [21]. This study was undertaken to evaluate the effects of a crude extract of *Caenorhabditis elegans* (CEC) on the development of OVA-induced asthma in a murine model. We found that CEC strongly suppressed airway inflammation not only during the development of asthma but also after the establishment of asthma in mice and that IFN-γ was the most important cytokine in the CEC-mediated suppression of asthma. This study provides new insights for understanding immune modulation by *C. elegans*-derived products as well as the potential application of these products as a modality for asthma treatment.

## Materials and Methods

### Ethics Statement

The protocols of animal experiments were reviewed and approved by the Institute of Laboratory Animal Resources of Seoul National University (Permit Number: SNU-080718-1, SNU-081008-2 and SNU-090624-9).

### Animals

Seven-week-old female BALB/c mice were used in this study. Wild-type BALB/c mice were obtained from KoaTech (Gyonggi-do, Korea), and IFN-γ knockout (KO) mice on a BALB/c background were purchased from the Jackson Laboratory (Bar Harbor, ME, USA). The animals were maintained at the clean barrier animal facilities at the Seoul National University College of Medicine under specific pathogen-free conditions.

### Experimental groups

The animals were divided into three to five groups, depending on the experimental protocol. Each group consisted of five mice. To investigate the effects of CEC on the development of asthma, mice were divided into the following three groups: a phosphate-buffered saline (PBS)-treated group (PBS), an OVA-treated group (OVA) and a group of mice sensitized with OVA and CEC (OVA+CEC) (Exp. 1). For the experiment designed to observe the role of IFN-γ in the effect that CEC has on asthma development, IFN-γ KO and wild-type mice were each divided into the following two groups: an OVA-treated group and an OVA+CEC-treated group (Exp. 2). The effects of CEC were also investigated in mice with established asthma using a different CEC treatment regimen and the experimental groups were as follows: a PBS-challenged group, an OVA-challenged group, groups challenged with a single dose of 50 µg (CEC M50) or 100 µg (CEC M100) or 25 µg weekly for one month (CEC W25) (Exp. 3).

### Preparation of *C. elegans* crude extract

The *C. elegans* N2 strain was grown in media supplemented with *Escherichia coli* OP50 as a food source. The worms were incubated at room temperature for six days. Adult worms were isolated, washed three times in sterile distilled water and homogenized in PBS with a sonicator while on ice. Following the centrifugation of the worm homogenate, the supernatant was passed through a 0.45-µm filter for sterilization and was then used as the crude extract of *C. elegans*. To eliminate endotoxin contamination, CEC preparations were passed through Detoxi-Gel Endotoxin Removing Gel AffinityPak pre-packed columns (Thermo Scientific Pierce Protein Research Products, Rockford, IL, USA).

### Sensitization and challenge protocol

For Exp. 1 and 2, mice in the OVA group were sensitized with 75 µg OVA (Sigma-Aldrich, St Louis, MO, USA) in 3 mg aluminum hydroxide (alum, Sigma-Aldrich) by intraperitoneal injections on days 0 and 7. The OVA+CEC group was sensitized with 10 µg CEC together with alum-precipitated OVA, and the PBS group was sensitized with PBS-alum. On days 14, 15, 21 and 22, mice were intranasally challenged with 50 µg OVA. On day 23, AHR was determined after the mice were anesthetized with an intraperitoneal injection of zoletile (Virbac Laboratories, Carros, France). Mice were sacrificed for the collection of BAL fluid, sera and lungs on day 24.

For Exp. 3, asthma was induced in mice by sensitization and challenge with OVA using the same protocol as Exp. 1, except for PBS group sensitized and challenged with PBS. AHR was measured after the last challenge with OVA at week 3 to confirm the establishment of asthma. Following stabilization, the mice were divided into four groups and intranasally challenged with either PBS, OVA or CEC at various doses and intervals: i.e., a single dose of 50 µg (CEC M50) or 100 µg (CEC M100) or 25 µg weekly for one month (CEC W25). At week 8, the methacholine-induced AHR was measured, and then the mice were sacrificed for serum and BAL collection.

### Airway hyperresponsiveness measurement

The methacholine (acetyl-β-methyl-choline chloride; Sigma)-induced AHR for Exp. 1 was evaluated using a computer-controlled animal ventilator system after the last challenge with OVA, as previously described [24]. Mice were anesthetized with zoletile, tracheostomized and connected to the flexiVent system (SCIREQ, Montreal, QC, Canada). Baseline lung mechanics were measured following the ultrasonic nebulization of PBS into the airways, and increasing does of methacholine (6.25, 12.5 and 25 mg/ml) were subsequently nebulized into the lungs of mice to measure AHR.

For Exp. 2 and 3, the methacholine-induced AHR was assessed using a whole-body plethysmograph (Allmedicus, Anyang, Gyonggi-do, Korea) 24 hr after the final challenge with OVA, as previously described [25]. Enhanced pause (Penh) values were measured by nebulizing methacholine into the airways at concentrations of 6.25, 12.5 and 25 mg/ml.

### Bronchoalveolar lavage and total and differential cell counts

The tracheas of anesthetized mice were cannulated, and the lungs were lavaged three times with 0.5 ml sterile PBS. A total of 1.4 ml BAL fluid was collected and centrifuged at 400× g for 10 min at 4°C. The supernatants were stored for subsequent cytokine assays, and the cell pellets from the BAL fluid were suspended in 100 µl PBS for the total and differential cell counts. The total number of inflammatory cells in the BAL fluid was counted using a hemocytometer. For the differential counts, the cells in the BAL fluid were centrifuged onto slides, and the cytospin preparations were stained with Giemsa. One hundred cells were counted in randomly selected fields, and differential cell counts were presented as an average of three independent counts.

### Lung histology

The lungs were removed from the mice following the collection of the BAL fluid and were then fixed in 10% phosphate-buffered formalin for 24 hr. The tissues were embedded in paraffin and cut into 3–5 µm sections. Histopathological observations were made by light microscopy after the sections were stained with hematoxylin and eosin.

### Cytokine analysis

The cytokine levels in the BAL fluid were measured using commercial sandwich ELISA kits for IL-4, IL-5, IL-10, IL-12p70, IL-13 and IFN-γ, according to the manufacturer's instructions (eBioscience, Inc., San Diego, CA, USA). The detection limits for each cytokine were 4 pg/ml for IL-4, IL-5 and IL-13, 15 pg/ml for IL-12p70 and IFN-γ and 30 pg/ml for IL-10.

### Measurement of serum antibody levels

Blood was drawn from anesthetized mice by cardiac puncture, and sera were collected and stored at −20°C until use. The serum levels of the total and OVA-specific IgE and IgG subtypes were measured by sandwich ELISA, according to the manufacturer's instructions (Bethyl Laboratories, Inc., Montgomery, TX, USA). The IgE, IgG1 and IgG2a levels were determined based on standard curves generated from known amounts of mouse IgE or IgG (PharMingen, San Diego, CA, USA). The detection limits were 0.1 ng/ml for IgE and 1 ng/ml for IgG.

### Statistical analysis

All data were expressed as the mean ± SD, and statistical significance for comparisons between groups was determined using Student's *t*-test. *P*-values less than 0.05 were considered statistically significant.

## Results

### CEC inhibits the development of OVA-induced airway inflammation and airway hyperresponsiveness

**Figure 1:**
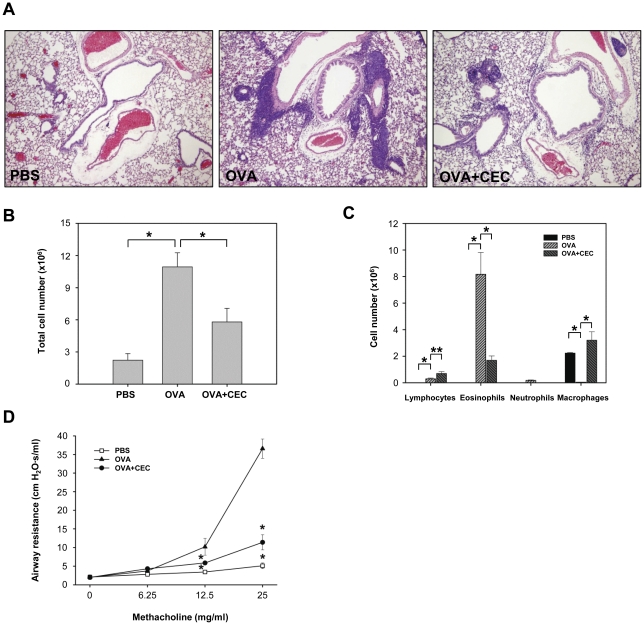
The suppressive effect of the crude extract of *Caenorhabditis elegans* (CEC) on the development of airway inflammation. Mice were sensitized with PBS, OVA or OVA+CEC by intraperitoneal injections on days 0 and 7, and then intranasally challenged with OVA on days 14, 15, 21 and 22. AHR was determined on day 23 and mice were sacrificed for the collection of BAL fluid, sera and lungs on day 24. (A) Histopathological observation of the lungs. H&E, ×100. (B) Total cell counts in the BAL fluid. (C) Differential cell counts of BAL cells. (D) Airway hyperresponsiveness to increasing concentration of methacholine presented as airway resistance. Data are presented as the mean ± SD from a single experiment representative of three separate experiments. PBS (n = 5), mice sensitized with PBS and challenged with OVA; OVA (n = 5), mice sensitized and challenged with OVA; OVA+CEC (n = 5), mice sensitized with OVA and CEC and challenged with OVA.*, *P*<0.01. **, *P*<0.05.

The airway inflammatory responses to OVA with or without CEC treatment were evaluated in a murine model of asthma. The intense infiltration of inflammatory cells, predominantly eosinophils, into the lungs was observed near the bronchioles and vessels of mice sensitized and challenged with OVA (Figure 1A). In contrast, no inflammatory cell infiltration was observed in the lungs of mice both immunized and challenged with PBS only. When mice were sensitized with CEC together with OVA, the cellular infiltration into the lungs was dramatically reduced compared to the infiltration observed in the OVA group (Figure 1A).

The total cell counts in the BAL fluid were consistent with the histopathological findings of airway inflammation in the experimental groups. The mice in the OVA group showed the highest total cell counts in the BAL fluid, and the majority of the inflammatory cells were eosinophils (Figure 1B, C). However, the total number of cells in the BAL fluid of the OVA+CEC group was significantly decreased in comparison to the OVA group (*P*<0.01) (Figure 1B, C). The eosinophil counts in the BAL fluid were significantly lower in the CEC-treated group than in the OVA group (*P*<0.01) but were still elevated compared to those in the PBS group. In contrast, the macrophage counts were significantly higher in the OVA+CEC group as compared to the OVA group (*P*<0.01), but they were not significantly different from the PBS group (P = 0.2209) (Figure 1C).

The OVA-sensitized mice showed a significant dose-dependent increase in methacholine-induced AHR compared with the PBS-treated control mice (*P*<0.01) (Figure 1D). However, this increase in methacholine-induced AHR in the OVA group was significantly suppressed by CEC sensitization (*P*<0.01) (Figure 1D).

### CEC treatment decreases allergen-induced Th2 cytokine production but increases Th1 cytokine levels in BAL fluid

**Figure 2:**
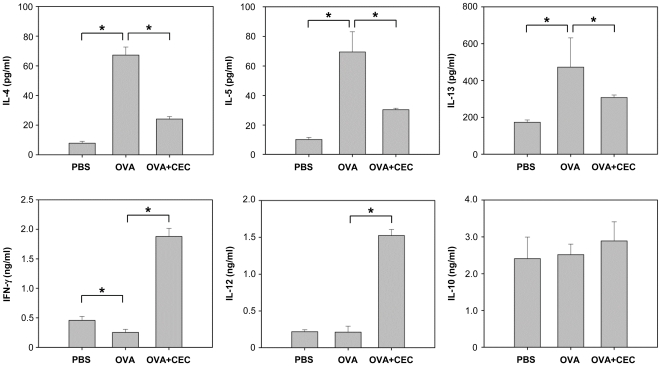
Cytokine analysis of the BAL fluid. Data from individual mice are presented as arithmetic means in histograms. PBS (n = 5), mice sensitized with PBS and challenged with OVA; OVA (n = 5), mice sensitized and challenged with OVA; OVA+CEC (n = 5), mice sensitized with OVA and CEC and challenged with OVA. *, *P*<0.01.

We investigated the cytokine milieu of the lungs to understand the mechanism by which CEC suppresses allergic airway inflammation. The mice sensitized with OVA showed significantly increased levels of the Th2 cytokines IL-4, IL-5 and IL-13 in comparison to the PBS group (*P*<0.01). But the IFN-γ level of the OVA group was significantly lower than that of the PBS group (*P*<0.01), and the IL-12 levels were equivalent between groups (Figure 2). However, the addition of CEC treatment resulted in a significantly reduced level of Th2 cytokines, as compared to the OVA group (*P*<0.01), whereas the levels of Th1 cytokines such as IFN-γ and IL-12 were significantly higher in the OVA+CEC group than the PBS and OVA groups (*P*<0.01) (Figure 2). In contrast, no difference in IL-10 production was found between the OVA and OVA+CEC groups (Figure 2).

### CEC treatment suppresses IgE and IgG1 levels but enhances IgG2a levels in serum

**Figure 3:**
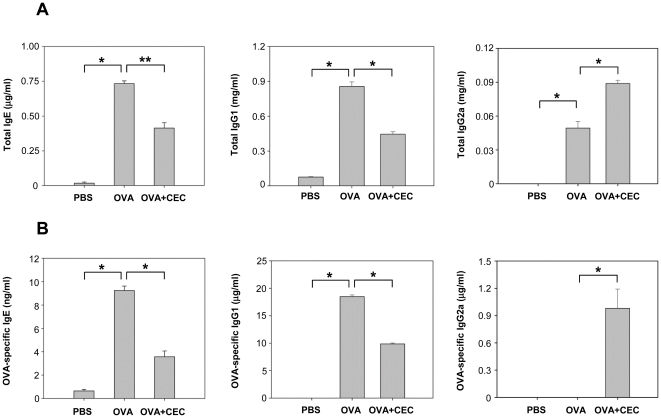
The evaluation of serum antibody titers in mice with OVA-induced airway inflammation. The production of total (A) and OVA-specific IgE, IgG1 and IgG2a (B) was evaluated to identify the effects of the *C. elegans* crude extract. Data from individual mice are presented as arithmetic means in histograms. PBS (n = 5), mice sensitized with PBS and challenged with OVA; OVA (n = 5), mice sensitized and challenged with OVA; OVA+CEC (n = 5), mice sensitized with OVA and CEC and challenged with OVA. *, *P*<0.01. **, *P*<0.05.

The serum levels of IgE, IgG1 and IgG2a were measured by sandwich ELISA. Both the total IgE and OVA-specific IgE responses were elevated in mice in the OVA group, as compared to those in the PBS or OVA+CEC groups (Figure 3). However, CEC treatment significantly diminished the allergic antibody responses in the OVA+CEC group compared to the OVA-group (total IgE, *P*<0.05; OVA-specific IgE, *P*<0.01) (Figure 3). Similar patterns were observed regarding the serum IgG1 levels, and the total IgG1 and OVA-specific IgG1 levels were significantly higher in the OVA group than in the PBS or OVA+CEC groups (*P*<0.01) (Figure 3). In contrast, the IgG2a level of the OVA+CEC group was significantly higher than that of the OVA group (*P*<0.01) (Figure 3).

### IFN-γ plays an important role in the suppressive effect that CEC sensitization has on asthma development

**Figure 4:**
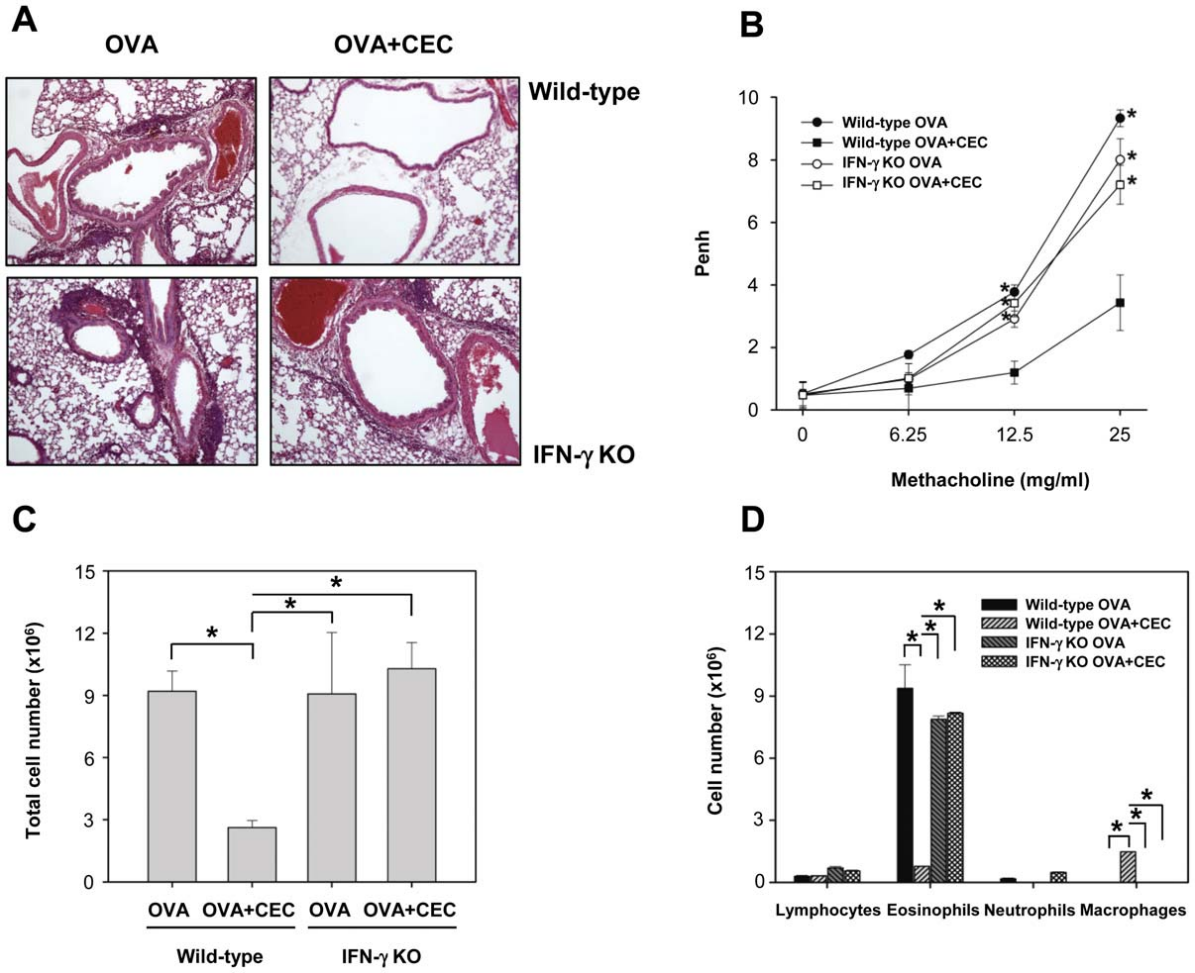
The role of IFN-γ in the CEC-mediated suppression of airway inflammation induced by OVA challenge in wild-type and IFN-γ KO mice. Mice were sensitized with PBS, OVA or OVA+CEC on days 0 and 7, and then challenged with OVA on days 14, 15, 21 and 22. AHR was determined on day 23, followed by sacrifice of mice for the collection of BAL fluid, sera and lungs on day 24. The infiltration of inflammatory cells into the lung (A), methacholine-induced airway hyperresponsiveness (B), and total (C) and differential cell counts (D) in the BAL fluid were investigated. Wild-type OVA (n = 5), wild-type mice sensitized with OVA; Wild-type OVA+CEC (n = 5), wild-type mice sensitized with OVA and CEC; IFN-γ KO OVA (n = 5), IFN-γ KO mice sensitized with OVA; IFN-γ KO OVA+CEC (n = 5), IFN-γ KO mice sensitized with OVA and CEC. *, *P*<0.01.

**Figure 5:**
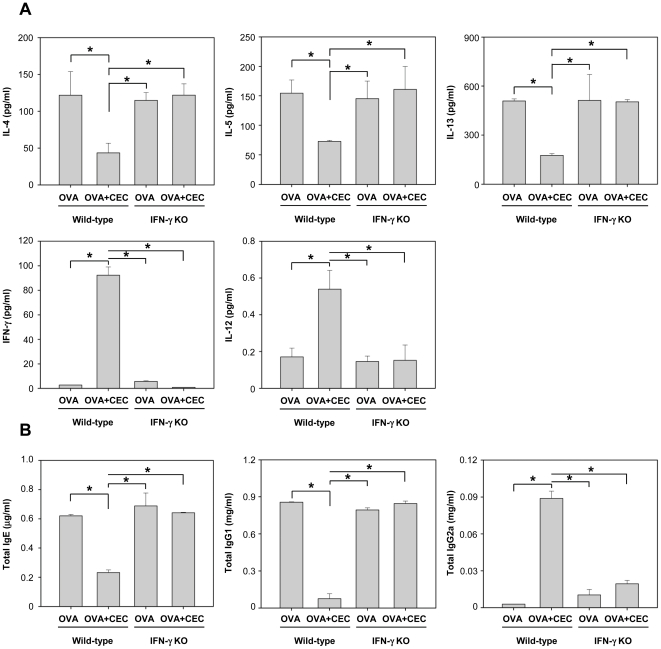
The evaluation of cytokine secretion in the BAL fluid (A) and the antibody titers from the sera (B) of wild-type and IFN-γ KO mice. Wild-type OVA (n = 5), wild-type mice sensitized with OVA; Wild-type OVA+CEC (n = 5), wild-type mice sensitized with OVA and CEC; IFN-γ KO OVA (n = 5), IFN-γ KO mice sensitized with OVA; IFN-γ KO OVA+CEC (n = 5), IFN-γ KO mice sensitized with OVA and CEC. *, *P*<0.01.

To elucidate the role of IFN-γ in the suppressive effect of CEC on the development of asthma, IFN-γ KO and wild-type mice were sensitized with OVA or OVA+CEC and challenged with OVA using the same protocol as previously described (Exp. 2). Increased airway inflammation, as characterized by AHR and the total cell counts of the BAL fluid including a high number of eosinophils, was evident in wild-type mice that had been sensitized with OVA, but these effects were significantly decreased in mice that had been treated with CEC (Figure 4) (*P*<0.01). However, in IFN-γ KO mice, the suppressive effect of CEC was abolished, and a similar degree of airway inflammation as that observed in wild-type mice sensitized with OVA was observed in these KO mice following OVA exposure (Figure 4). CEC sensitization suppressed the production of IL-4, IL-5 and IL-13 in wild-type mice but failed to suppress the production of these Th2 cytokines in IFN-γ KO mice (Figure 5A). In contrast, the levels of IFN-γ and IL-12 were significantly higher in wild-type mice sensitized with OVA+CEC than in mice of the other three groups (*P*<0.01) (Figure 5A). CEC sensitization significantly decreased the levels of total IgE and IgG1 but increased the level of total IgG2a in wild-type mice (*P*<0.01). However, the absence of IFN-γ resulted in increased levels of total IgE and IgG1 and decreased level of IgG2a despite CEC treatment (Figure 5B).

### CEC suppresses OVA-induced airway inflammation in established asthma

**Figure 6:**
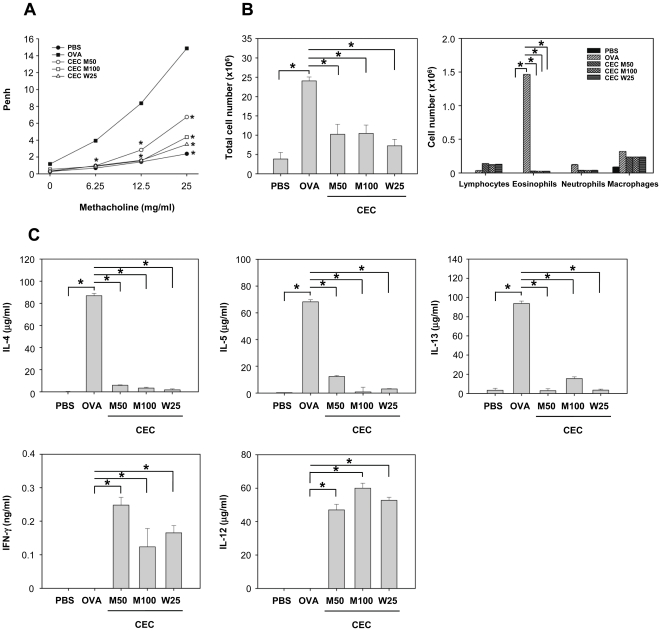
The suppressive effects of the crude extract of *Caenorhabditis elegans* (CEC) on established asthma in mice. Airway inflammation has been induced in mice by sensitization and challenge with OVA using the same protocol as in Fig. 1, whereas PBS group (n = 5), the negative control group, was sensitized and challenged with PBS. The establishment of asthma was confirmed by measuring AHR after the last challenge with OVA at week 3, and thereafter, mice were divided into four experimental groups and challenged with either OVA or various CEC regimens. PBS group and OVA group (n = 5) were intranasally challenged with PBS or OVA, respectively, at week 4 and 6. CEC M50 (n = 5) and CEC M100 (n = 5) groups were intranasally challenged once with a single dose of 50 µg or 100 µg CEC at week 4. CEC W25 (n = 5) group was intranasally challenged with 25 µg of CEC weekly for one month from week 4 to week 7. At week 8, the methacholine-induced AHR was measured, and then the mice were sacrificed for serum and BAL collection. (A) Airway hyperresponsiveness to methacholine was measured using a whole-body plethysmograph at week 8. Data are presented as the mean ± SD from a single experiment representative of three separate experiments. (B) Total and differential cell counts in the BAL fluid. (C) Analysis of cytokines in the BAL fluid. Data from individual mice are presented as arithmetic means in histograms. *, *P*<0.01.

The suppressive effect of CEC on established asthma was investigated using a different CEC treatment regimen during the 4 weeks after asthma had been established (Exp. 3). When mice were challenged with CEC after the establishment of asthma, the airway inflammation, including AHR and the total and differential cell counts in the BAL fluid, was significantly decreased in a dose-dependent manner (Figure 6A, B). Furthermore, an analysis of the cytokines in the BAL fluid showed a decreased Th2 cytokine response and an increased Th1 response following CEC challenge, whereas OVA-challenged mice showed a Th2-dominant cytokine response (Figure 6C).

## Discussion

Epidemiological and experimental studies have shown the protective effects that helminth infections have against asthma in humans and animals. In particular, infection by intestinal nematodes and schistosomes has been shown to be beneficial for allergic diseases in humans [2], [26]. Based on this, clinical trials using helminths to treat immunological diseases in humans have been implemented [16], [27]. Helminth therapy using the eggs of *Trichuris suis*, a pig whipworm, has been effective for the treatment of inflammatory bowel diseases such as ulcerative colitis and Crohn's disease [18], [19]. Furthermore, a hookworm infection intervention study showed improved AHR among asthma patients as compared to those in the placebo group, although this difference was not significant [17]. However, the use of worms for human treatment is not be entirely harmless, as one patient with Crohn's disease was shown to have developed an iatrogenic infection of *T. suis* [28].

*C. elegans* was selected as a model organism in this study to observe the effects it could have on the suppression of allergic airway inflammation in a murine model of asthma. Because of its well-known biology and completely sequenced genome, *C. elegans* is one of the most versatile organisms for study in biomedical fields such as neuroscience and developmental biology [29]. It has a short lifespan of approximately 2–3 weeks and is easily cultivated in large numbers in liquid media or on agar plates with *E. coli*. Although *C. elegans* is a free-living, non-parasitic nematode, it could potentially induce a host immune response similar to that induced by other intestinal nematodes, if given the chance to enter a host. Cystatins of *C. elegans* has been shown to increase the production of IFN-γ and IL-12 by human peripheral blood mononuclear cells [30], and they can inhibit cathepsin B, leading to enhanced expression of Th1 cells [31]. This finding suggests that components of *C. elegans* may have an inhibitory effect on allergic airway inflammation by enhancing the Th1 responses.

As *C. elegans* was cultured in media supplemented with *E. coli*, CEC samples were flowed through endotoxin removing gels to remove endotoxin by affinity chromatography, and the endotoxin level in 10 µg CEC for sensitization of mice was determined to be less than 1 endotoxin unit/mg. In our preliminary study, dendritic cells stimulated in vitro by CEC produced higher amount of IL-12 than by LPS alone or together with CEC, and CEC induced IL-12 and IFN-γ production in naïve mice (data not shown). Therefore, it is considered that CEC can induce Th1 responses in mice, and the amount of possible contamination of LPS in CEC samples is negligible to have effects seen in this study.

Because asthma is associated with dysregulated Th2 responses, enhanced Th1 responses may suppress the development of allergic airway inflammation [21]. Therefore, strategies that enhance Th1 responses have been proposed as therapies for ameliorating allergic airway inflammation. The administration of IFN-γ into the airways by nebulization inhibits the development of AHR, OVA-specific IgE responses and cutaneous reactivity to OVA, and this administration also prevents the development of secondary allergen sensitization even after primary allergic responses are well established in mice [32]. Although airway inflammation and AHR persist for 8 weeks following OVA challenge in IFN-γ KO mice, 1 week of IFN-γ treatment is able to reverse this asthmatic reaction, even when the allergic airway reaction is established [33]. In the present study, the attenuation of airway inflammation and AHR by CEC treatment either during the development of or after the establishment of asthma was associated with a shift from a Th2 to a Th1 response in mice sensitized and challenged with OVA. Moreover, the decreased production of Th2 cytokines and allergen-specific antibodies and the drastically increased concentrations of Th1 cytokines, such as IFN-γ and IL-12, were also observed in the BAL fluid. Importantly, IFN-γ KO mice exhibited no attenuation in airway inflammation following CEC treatment, suggesting that IFN-γ plays an essential role in the CEC-mediated inhibition of allergic airway inflammation. Therefore, the Th2-dominant cytokine response resulting from OVA sensitization and challenge in mice was altered to a Th1-dominant response by CEC treatment, and this treatment led to the inhibition of asthma in this study. However, heat-inactivated CEC did not inhibit allergic airway inflammation, suggesting that the properties of CEC in suppression of asthma in mice are proteinaceous (data not shown).

In CEC-treated BALB/c mice, the number of alveolar macrophages was significantly increased in comparison to the group that only received OVA (Figure 1C). Alveolar macrophages, which are the most abundant haematopoietic cells in the lung, play an important role in pulmonary innate immunity. However, they have also been suggested to suppress allergic asthma by secreting soluble molecules such as IFN-γ and IL-12 and/or by directly interacting with other cells [34]. The depletion of alveolar macrophages in OVA-sensitized mice was shown to markedly enhance airway inflammation and AHR, and the adoptive transfer of OVA-pulsed lung macrophages selectively promoted a Th1 response [35]. In the present study, the concentration of IL-12 and the number of macrophages in the BAL fluid of OVA-challenged wild-type mice were increased following CEC treatment. Moreover, IFN-γ deficiency of the OVA-challenged hosts ablated these effects. Therefore, these results suggest that CEC-induced Th1 responses may suppress the development and progression of asthma by activating alveolar macrophages and thereby enhancing their production of IL-12. Although the mechanism by which CEC treatment enhances Th1 responses has not been fully investigated, CEC seems to modulate the Th1/Th2 balance by affecting the function of dendritic cells (DCs), as DCs cultured in the presence of CEC produced a higher amount of IL-12 than control DCs (data not shown).

Regarding the immunological mechanisms by which helminth infections suppress the development of allergic responses, it has been proposed that changes in the regulatory networks of IL-10 or regulatory T cells (Treg cells) as well as the induction of alternatively activated macrophages or immunosuppressive B cells are involved [1]. For example, cystatin, the secreted product of the rat filarial nematode *Acanthocheilonema viteae*, was shown to protect mice from allergic airway inflammation and dextran sulfate sodium-induced colitis, and this was likely due to enhanced IL-10 production by macrophages but not Treg cells [36]. IL-10 mediated suppression of allergen-induced airway eosinophilia was observed in mice infected with *N. brasiliensis* 4 or 8 weeks before OVA-airway challenge [13]. However, IL-10 may not play a major role in our system, as no significant increase in IL-10 levels in the BAL fluid of CEC-treated mice was observed. However, further study is needed to clarify the role of Treg cells in the CEC-mediated inhibition of asthma development.

This study clearly demonstrated that crude extracts of *C. elegans* inhibited the development of allergen-specific Th2 responses by shifting the Th2 response to a Th1 response and that IFN-γ plays an important role in the CEC-mediated amelioration of both acute and established asthma. However, the Th1/Th2 balance may be differentially modulated depending on the types of parasite antigens present. ES-62, a phosphorylcholine-containing glycoprotein of *A. viteae*, is the best-characterized immunoregulatory molecule derived from helminths [1], [20] and has been shown to suppress airway inflammation in mice [37] and decrease the severity and progression of collagen-induced arthritis by inhibiting collagen-specific pro-inflammatory/Th1 cytokine release [38]. In contrast, *Ascaris suum* contains two different protein components that have opposite effects on experimental asthma in mice [39]. The allergenic protein of *A. suum* (APAS-3) induced Th2 responses, such as eosinophilic infiltration into the airways and the induction of IL-4, IL-5 and AHR, in BALB/c mice [39], whereas the suppressive protein of *A. suum* (PAS-1) markedly suppressed APAS-3-induced airway inflammation [39], [40]. The components of CEC that are responsible for the suppression of airway inflammation observed in this study have yet to be elucidated. The possible candidates may include cysteine protease inhibitors, chemokine binding proteins, phosphorylcholine-containing proteins, glycans and glycolipids [16], [36].

In this study, two methods, the non-invasive whole body plethysmography (Penh method) and the invasive technique using a computer-controlled animal ventilator (flexiVent system), were used to assess measure airway function in asthma-induced mice. With advantages of decreasing the cost, time and the number of animals required for experiments and the remarkable ease of use, the whole body plethysmography has been widely used in the measurement of lung function in mice [41], [42]. However, many evidences showed that Penh does not always correlate with airway resistance and therefore, Penh is required to be confirmed by invasive techniques [41], [42]. We found that Penh correlated well with airway resistance measured by flexiVent system in our model system of asthma in Exp. 1 (data not shown), and therefore, the whole body plethysmography was used to assess the airway responsiveness in Exp. 2 and 3.

To our knowledge, this is the first report to document the suppressive effect that *C. elegans*-derived extracts have on allergic airway inflammation in a murine model of asthma. However, one should be cautious when attempting to extrapolate data from animal models of asthma to the human disease, and the relevance of our observations in mice to human asthma remains to be completely determined. Moreover, the role of Th1 cytokines, especially IFN-γ, in the development of asthma is controversial, as conflicting evidence has suggested that it can both suppress and enhance Th2 responses in mouse models of allergic airway inflammation [22], [33], [43]. Nevertheless, the present study provides new insights into understanding the immune modulation governed by parasite-derived products and the development of new asthma treatment strategies.
